# Pegylated recombinant human granulocyte colony-stimulating factor for primary prophylaxis of neutropenia in patients with cervical cancer receiving concurrent chemoradiotherapy: a prospective study

**DOI:** 10.1186/s12885-024-12556-4

**Published:** 2024-07-12

**Authors:** Jing You, Yidi Yuan, Xiaoxuan Gu, Weihu Wang, Xiaofan Li

**Affiliations:** Key Laboratory of Carcinogenesis and Translational Research (Ministry of Education/Beijing), Department of Radiation Oncology, Peking University Cancer Hospital & Institute, No. 52 Fucheng Road, Haidian District, Beijing, China

**Keywords:** Colony-stimulating factor, Neutropenia, Cervical cancer, Chemoradiotherapy

## Abstract

**Background:**

This study aimed to investigate the efficacy and safety of pegylated recombinant human granulocyte colony-stimulating factor (PEG-rhG-CSF) for primary prophylaxis of neutropenia in patients with cervical cancer receiving concurrent chemoradiotherapy.

**Methods:**

In this prospective, single-center, single-arm study, we enrolled patients (18–70 years) with 2018 International Federation of Gynecology and Obstetrics (FIGO) stage IIIC1r-IVA and IVB (distant metastasis only with inguinal lymph node metastasis) cervical cancer. Eligible patients should have normal function of the bone marrow (absolute neutrophil count (ANC) ≥ 2.0 × 10^9^/L) and adequate hepatic and renal functions. Key exclusion criteria included: previous chemotherapy and/or radiotherapy; a history of bone marrow dysplasia or other hematopoietic abnormalities. All patients underwent radical radiotherapy (pelvic radiotherapy or extended-field irradiation) plus brachytherapy. The chemotherapy regimen included four cycles of 3-weekly paclitaxel and cisplatin. PEG-rhG-CSF was administered 48–72 h after each treatment cycle. Salvage granulocyte colony-stimulating factor (G-CSF) was only permitted in certain circumstances. The primary endpoint was the incidence of grade 3–4 neutropenia. The secondary endpoints included frequency of febrile neutropenia (FN), chemotherapy completion rate in cycles 2–4, time to complete radiotherapy, and safety.

**Results:**

Overall, 52 patients were enrolled in this study from July 2019 to October 2020. The incidence of grade 3–4 neutropenia was 28.8%, with an average duration of grade 3–4 neutropenia persistence of 3.85 days (1–7 days). The incidence rate of FN was 3.8%. The chemotherapy completion rate was 94.2%, 82.7%, and 75.0% for cycles 2–4, respectively. The incidences of grade 3–4 neutropenia for cycles 1–4 were 9.6% (5/52), 8.2% (4/49), 14.0% (6/43), and 2.6% (1/39), respectively. All patients completed radiotherapy within 8 weeks (median, 48 days; range: 41–56 days), except one patient who withdrew consent and did not receive radiotherapy. Severe non-hematologic toxicity was not observed in any patient.

**Conclusion:**

PEG-rhG-CSF is an effective and safe prophylactic treatment for neutropenia in patients with cervical cancer undergoing concurrent chemoradiotherapy.

**Trial registration:**

Chinese Clinical Trial Registry, ChiCTR1900024494. Date of Registration:13/July/2019.

## Introduction

According to the 2015 edition of cancer statistics in China, cervical cancer is the most commonly diagnosed genitourinary malignant tumor and the leading cause of cancer-related deaths in Chinese women [1]. The addition of concurrent cisplatin-based chemotherapy to definitive radiotherapy has been the standard treatment for locally advanced cervical cancer since 1999 [2, 3]. Nevertheless, the incidence of distant metastasis is high, and it has become the predominant treatment failure pattern [4, 5]. In 2011, a randomized trial reported a survival advantage with the addition of gemcitabine to concurrent chemoradiotherapy (CCRT), followed by adjuvant chemotherapy [6]. Further, a meta-analysis including 13 trials demonstrated that radiotherapy concurrent with platinum-based doublet therapy improved survival compared to single-agent cisplatin therapy [7]. Despite the potential benefits of this chemotherapy regimen, the high incidence of myelotoxicity, especially neutropenia, limits its use in clinical practice.

Febrile neutropenia (FN) is mainly defined as the concurrence of grade 3–4 neutropenia and fever, which is a major obstacle to achieving full-dose chemotherapy. A previous study has shown that the prophylactic application of granulocyte colony-stimulating factor (G-CSF) can significantly reduce the incidence of FN and the rate of infection-related mortality and improve the relative dose intensity (RDI) of chemotherapy [8]. In a systematic review of 25 clinical trials, including over 12,000 patients, these benefits translated to survival advantages, with a 3.4% reduction in the absolute mortality risk [9].

Based on the current National Comprehensive Cancer Network (NCCN) guidelines [10], the regimen of paclitaxel and cisplatin for cervical cancer is considered an intermediate risk factor for FN, with an incidence of 10–20%. In this case, prophylactic use of G-CSF was recommended when patients had at least one risk factor, including previous radiotherapy. In the clinical practice, neutropenia is more common than FN and worthy of note. In a trial investigating the efficacy of weekly cisplatin and paclitaxel concurrent with radiotherapy in locally advanced cervical caner patients, one major reason for patients failing to complete the scheduled chemotherapy cycles was neutropenia [11].

The covalent combination of polyethylene glycol (PEG) extends the half-life of G-CSF from 3 to 4 h to approximately 42 h, allowing it to be administered only once per cycle of chemotherapy instead of daily administration [12]. Moreover, a randomized trial demonstrated that pegylated recombinant human granulocyte colony-stimulating factor (PEG-rhG-CSF) achieved at least the same effect as that of G-CSF [13]. Thus, prophylactic use of PEG-rhG-CSF instead of G-CSF brings benefits of fewer injections, less travel burden and better patient compliance. However, most studies demonstrated these potential benefit in patients receiving multiple cycles of chemotherapy, supporting evidence of its use during CCRT is still limited. Therefore, we conducted this study to evaluate the efficacy and safety of PEG-rhG-CSF for the primary prevention of neutropenia in cervical cancer patients treated with CCRT.

## Materials and methods

### Patients and study design

In this prospective, open-label, single-arm clinical trial, we assessed the efficacy and safety of PEG-rhG-CSF as primary prophylaxis for neutropenia during CCRT in patients with cervical cancer. The inclusion criteria were as follows: (1) newly diagnosed cervical squamous cell carcinoma, adenocarcinoma, or adenosquamous cell carcinoma; (2) 2018 International Federation of Gynaecology and Obstetrics (FIGO) stage IIIC1r-IVA or IVB (distant metastasis only with inguinal lymph node metastasis); (3) age 18–70 years; (4) no history of hematological disease; (5) normal function of the bone marrow (absolute neutrophil count (ANC) ≥ 2.0 × 10^9^/L, hemoglobin level ≥ 80 g/L, platelet count ≥ 90 × 10^9^/L); (6) adequate hepatic and renal functions; and (7) Karnofsky performance status score ≥ 70. Key exclusion criteria included the following: previous chemotherapy and/or radiotherapy; uncontrolled infection; bone marrow dysplasia or other hematopoietic abnormalities; pregnancy or lactation; a history of other malignancy. This study was registered in the Chinese Clinical Trial Registry as ChiCTR1900024494, 13/07/2019.

### Treatment protocol

The patients were scheduled to undergo four cycles of chemotherapy. The first and second cycles were concurrent with radiotherapy, and the third and fourth cycles were adjuvant chemotherapy. In patients with bulky tumors, induction chemotherapy was initiated during the first cycle. In this case, the second and third cycles were concurrent with radiotherapy, and the fourth cycle was adjuvant chemotherapy. The induction chemotherapy and adjuvant chemotherapy regimen both comprised paclitaxel 175 mg/m^2^ on day 1 and cisplatin 75 mg/m^2^ divided into 2 days on days 1–2 with an interval of 21 days between them. During CCRT, the paclitaxel and cisplatin levels were reduced to 135 mg/m^2^ and 60 mg/m^2^(divided into 2 days on days 1–2), respectively.

The external beam radiation dose for the pelvic clinical target volume (CTV) was 45 Gy delivered in 25 fractions over 5 weeks. The target dose for the involved metastatic lymph nodes was increased to 60 Gy using a simultaneous integrated boost technique. After external beam radiotherapy, an additional dose of 6–10 Gy was considered for the residual lymph nodes. Patients with common iliac and/or para-aortic lymph node involvement were treated with extended-field radiotherapy including simultaneous radiotherapy of pelvic CTV and the para-aortic CTV. The para-aortic CTV was extended from the upper border of pelvic CTV to the level of the left renal vein. The pelvic and vertebrae bone marrow was contoured on the planning computed tomography scan to limit radiotherapy-related myelotoxicity, and the dose constraints were both Dmean ≤ 25 Gy. Brachytherapy was performed in the fourth or fifth week after initiating external irradiation using a high-dose-rate 192 Ir afterloader. For patients receiving CT-based image-guided adaptive brachytherapy (IGABT), the D90 high-risk clinical target volume was at least 85 Gy. For patients receiving two-dimensional brachytherapy, the total dose (including external beam radiation and brachytherapy) to point A should exceed 85 Gy.

For patients with a body weight of ≥ 45 kg, a fixed 6-mg PEG-rhG-CSF (CSPC Pharmaceutical Group Co., Ltd., Wanchai, Hong Kong) was administered subcutaneously 48–72 h after each cycle of chemotherapy. The dose of PEG-rhG-CSF was reduced to 3 mg for patients with a body weight of < 45 kg. Salvage treatment for neutropenia was conducted as follows: during CCRT, 5 µg/kg/d G-CSF was injected until ANC was ≥ 5.0 × 10^9^/L when patients experienced: (1) ANC ≤ 1.0 × 10^9^/L and/or FN was observed within 14 days of PEG-rhG-CSF administration; (2) ANC ≤ 1.5 × 10^9^/L and/or FN after 14 days of administration. During induction and adjuvant chemotherapy, salvage G-CSF was only permitted when ANC was ≤ 0.5 × 10^9^/L and/or FN was observed. The prophylactic use of antibiotics was prohibited during the study, except for that in cases of FN, infection, or body temperature ≥ 38℃ with suspected infection.

Complete blood counts (CBC) were collected on days 8, 11 ,14 and 21 for cycles 1–3, and on days 15 for cycle 4. The frequency of CBC could be increased if necessary.

### Study endpoints and statistical analysis

The primary endpoint was the incidence of grade 3–4 neutropenia (ANC < 1.0 × 10^9^/L). At our center, the incidence of grade 3–4 neutropenia in patients with cervical cancer receiving radiotherapy concurrently with paclitaxel and cisplatin is approximately 50%. We expected the proportion of patients developing grade 3–4 neutropenia to reduce to 30% with PEG-rhG-CSF support. With a power of 80% and α level of 5% (two-sided test), the minimum sample size was 47 patients. Considering a 10% loss to follow-up rate, the number of participants was 52. The PASS 15.0 software (NCSS, LLC, Kaysville, UT, USA) was used to calculate the sample size for this study.

The secondary endpoints were frequency of FN, rate of chemotherapy completion in cycles 2–4, time to complete radiotherapy, and safety. FN was defined according to NCCN guidelines based on the following two criteria: (1) single oral temperature ≥ 38.0 °C for over 1 h or ≥ 38.3 °C; (2) ANC ≤ 0.5 × 10^9^/L or ≤ 1.0 × 10^9^/L with a tendency of dropping below 0.5 × 10^9^/L over the following 48 h. Toxicities were evaluated based on the Common Terminology Criteria for Adverse Events (CTCAE) version 4.0. Statistical analyses were performed using SPSS 25.0 software (SPSS, Inc., Chicago, IL, USA). Categorical variables are presented as percentages. Descriptive data are reported as median and range.

## Results

### Baseline characteristics

From July 16th, 2019 to October 27th, 2020, a total of 52 patients signed informed consent forms and were enrolled in the study. Detailed patient demographics are shown in Table 1. The median age at diagnosis was 50.9 years (range, 28–68 years). Most patients had squamous cell carcinoma (94.2%). Regarding the FIGO stage, 67.3% of patients had stage IIIC1r, 23.1% had stage IIIC2r, 3.8% had stage IVA, and 5.8% had stage IVB (only with inguinal lymph node metastasis). Most patients (78.8%) had bulky tumors (> 40 mm), and the median tumor size for the entire study population was 50.5 mm (range, 16–80 mm). Overall, 33 (63.5%) and 19 patients (36.5%) received extended-field irradiation and pelvic radiotherapy, respectively. Among the patients, 34.6% had anemia before treatment. The median pretreatment squamous cell carcinoma antigen level was 20.2 ng/mL (range, 0.8–131 ng/mL).

**Table 1:** Baseline characteristics of patients

Characteristics	*N* = 52
Age (years)	
Median (range)	50.9 (28–68)
<60	41 (78.8%)
≥ 60	11 (21.2%)
ECOG PS	
0	19 (36.5%)
1	33 (63.5%)
Pathologic type	
Squamous cell carcinoma	49 (94.2%)
Adenocarcinoma	1 (1.9%)
Adenosquamous carcinoma	2 (3.9%)
FIGO staging	
IIIC1r	35 (67.3%)
IIIC2r	12 (23.1%)
IVA	2 (3.8%)
IVB	3 (5.8%)
Tumor size (mm)	
Median (range)	50.5 (16–80)
≤ 40	11(21.2%)
> 40	41(78.8%)
Extended-field irradiation	
Yes	33 (63.5%)
No	19 (36.5%)
Brachytherapy	
2-dimensional brachytherapy	25 (48.1%)
CT-based IGABT	27 (51.9%)
Pretreatment hemoglobin (g/L)	
Median (range)	119.5 (80–159)
80–100	12 (23.1%)
100–110	6 (11.5%)
≥ 110	34 (65.4%)
Pretreatment SCC level (ng/mL)	
Median (range)	20.2 (0.8–131)
1.5–10	3 (5.8%)
10–70	45 (86.5%)
≥ 70	4 (7.7%)

ECOG PS, Eastern Cooperative Oncology Group Performance Status; FIGO, International Federation of Gynaecology and Obstetrics; IGABT, image-guided adaptive brachytherapy; SCC, squamous-cell carcinoma antigen

### Treatment completion

**Fig. 1:**
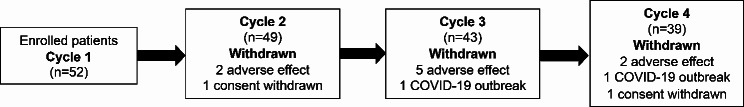
Flowchart of chemotherapy

The median number of chemotherapy cycles was four (range: 1–4) (Fig. 1). All patients received at least one cycle of chemotherapy with PEG-rhG-CSF. In total, 88.0% (183/208) of the scheduled chemotherapy cycles were completed. The chemotherapy completion rate was 94.2%, 82.7%, and 75.0% for cycles 2–4, respectively. The reasons for discontinuation of chemotherapy are listed in Table 2. Chemotherapy dose reduction occured in 6 patients (11.5%) for 8 cycles (4.4%). The reasons for dose reduction included grade 3 anemia, grade 4 thrombocytopenia, FN and grade 2 acute radiation-related toxicities. The mean RDI values for paclitaxel and cisplatin were 87.1% and 87.0%, respectively.

**Table 2:** Reasons for discontinued chemotherapy

Reasons	*N* = 13
Grade 3 anemia	4 (30.7%)
Continuous grade 2 neutropenia	4 (30.7%)
Grade 3 anemia with continuous grade 2 neutropenia	1 (7.8%)
COVID-19 outbreak	2 (15.4%)
Consent withdrawn	2 (15.4%)

In the study population, 20 patients (38.5%) underwent 23 cycles of delayed chemotherapy, and the reasons are summarized in Table 3. The main reasons for chemotherapy deferral were bone marrow suppression, including anemia (52.2%), neutropenia (30.4%), and thrombocytopenia (13.0%); the incidence of other causes was < 10%.

**Table 3:** Reasons for delayed chemotherapy

Reasons	*N* = 23
Grade 3 anemia	10 (43.5%)
Grade 3 anemia with grade 3 neutropenia	1 (4.3%)
Grade 2 anemia with vaginal bleeding	1 (4.3%)
Febrile neutropenia	2 (8.7%)
Continuous grade 2 neutropenia	2 (8.7%)
Grade 2 thrombocytopenia with grade 2 neutropenia	2 (8.7%)
COVID-19 outbreak	2 (8.7%)
Grade 2 thrombocytopenia	1 (4.3%)
Paroxysmal supraventricular tachycardia	1 (4.3%)
Pelvic infection	1 (4.3%)

Salvage G-CSF occured in 18 patients (34.6%) for 21 cycles (11.5%), including 9 cycles with grade 3–4 neutropenia within 14 days of administration PEG-rhG-CSF during CCRT, 9 cycles with grade 2 neutropenia after 14 days of administration during CCRT, 2 cycles with FN, and 1 cycle with grade 4 neutropenia during induction chemotherapy.

One patient withdrew consent after the first chemotherapy cycle and did not receive radiotherapy thereafter. The remaining patients (98.1%) completed external radiotherapy and brachytherapy within 8 weeks (median time, 48 days; range: 41–56 days).

### Incidence of grade 3–4 neutropenia and FN

The incidence of grade 3–4 neutropenia was 28.8%, with an average duration of grade 3–4 neutropenia persistence of 3.85 days (1–7 days). The incidences of grade 3–4 neutropenia for cycles 1–4 were 9.6% (5/52), 8.2% (4/49), 14.0% (6/43), and 2.6% (1/39), respectively. The incidence rate of FN was 3.8%. Two patients developed FN during CCRT: one patient after cycle 2 and the other after cycle 3.

### Other toxicities

Details on hematological and non-hematological toxicities are shown in Table 4. Anemia was the most common grade 3–4 toxicity except neutropenia (36.5%), followed by leukopenia (32.7%) and thrombocytopenia (15.4%). No cases of grade 3–4 acute proctitis or cystitis were observed. Mild-to-moderate bone pain was observed in 19.2% of the patients.

**Table 4:** Toxicities

	All grades *N* (%)	Grade 3 *N* (%)	Grade 4 *N* (%)
Leucopenia	42 (84.6%)	14 (26.9%)	3 (5.8%)
Neutropenia	33 (67.3%)	9 (21.1%)	4 (7.7%)
Anemia	48 (92.3%)	19 (36.5%)	0
Thrombocytopenia	40 (76.9%)	7 (13.5%)	1 (1.9%)
Febrile neutropenia	2 (3.8%)	-	-
Nausea and/or vomiting	50 (96.2%)	0	0
Neurotoxicity	20 (38.5%)	0	0
Bone pain	10 (19.2%)	0	0
Acute radiation enteritis	47 (90.4%)	0	0
Acute radiation cystitis	35 (67.3%)	0	0

The hospitalization rate was 23.1% (*n* = 12/52). Two patients with FN and one with a pelvic infection received antibiotics and supportive care. Seven patients with anemia and two with thrombocytopenia received blood transfusions.

## Discussion

In this prospective phase II study, we demonstrated that primary prophylactic PEG-rhG-CSF administered during CCRT in cervical cancer is well tolerated and that it reduces the incidence of severe neutropenia. Over the past two decades, progress in systemic therapy for locally advanced cervical cancer has been limited. The risk of developing distant metastases remains high, especially in patients with more advanced disease stages and metastatic lymph nodes [14]. Although the platinum-doublet regimen may have advantages in terms of survival over single-agent cisplatin, it is difficult to accomplish full-dose delivery of chemotherapy concurrent with radiotherapy. In a phase II trial, 56.8% of patients failed to complete the scheduled cycles, and the major reason was neutropenia [11]. Primary or secondary prophylaxis with G-CSF may be an effective solution to overcome this obstacle.

In a recently published study by Zou et al. [15], 60 patients with cervical cancer were randomized and divided into two groups in a 2:1 ratio. All patients were scheduled to undergo radiotherapy concurrently with two cycles of chemotherapy with paclitaxel and cisplatin. Additionally, patients in the experimental group received prophylactic PEG-rhG-CSF. This study showed that primary prophylaxis significantly reduced the incidence of grade 3–4 neutropenia from 77.78 to 10%. None of the patients in the experimental group experienced FN compared with the 16.67% of patients who did in the control group. With the support of PEG-rhG-CSF, the incidence of grade 3–4 neutropenia in our study was reduced from 50% (based on historical data of our center) to 28.8%. which is higher than that reported in the study by Zou et al. [15]. This difference can be attributed to two factors. First, patients were scheduled for two cycles of chemotherapy in the study by Zou et al., whereas we planned four cycles with the same chemotherapy regimen in our study. Second, patients enrolled in our study had more advanced stages (IIIC1r-IVB in our study compared to IIB-IIIB in the study by Zou et al.). Although the radiation field was not clearly described in the study by Zou et al., it is reasonable to speculate that more patients received extended-field irradiation in our study, which could also have resulted in more severe myelosuppression. The incidence of FN (3.8%) was also slightly higher in our study than that in the study by Zou et al.; however, it was acceptable.

Neutropenia was still a major reason (5 of 13 patients, 38.5%) for chemotherapy discontinuation. Notably, anemia was another predominant reason (38.5%) for chemotherapy intolerance. Anemia was correlated with more cases of delayed chemotherapy than neutropenia. Patients with bulky tumors are more likely to have vaginal bleeding, which can lead to anemia in some cases [16]. In this study, 78.8% of the enrolled patients had bulky tumors, and 34.6% had anemia before treatment. This may partly explain why anemia has a significant impact on chemotherapy compliance.

A systematic review including 25 randomized controlled trials demonstrated that the prophylactic use of G-CSF reduced all-cause mortality and that a greater survival benefit was associated with a higher chemotherapy dose intensity [9]. This result was consistent with the findings of another study, which found that G-CSF significantly improved the RDI after chemotherapy [8]. However, high-level evidence of a correlation between G-CSF application, RDI, and survival is limited for patients receiving chemoradiotherapy. A secondary analysis of the CONVERT trial explored the role of G-CSF during CCRT in small-cell lung cancer [17]. The optimal dose intensity of cisplatin and etoposide was achieved in significantly more patients in the prophylactic G-CSF group (both primary and secondary prophylaxis were included) than in the naive group. While the increased dose intensity of cisplatin was only associated with improved overall survival in the univariate analysis, there is still a possibility of a survival benefit when more patients receive primary prophylaxis with G-CSF. In our study, the mean RDI for paclitaxel and cisplatin were 87.1% and 87.0%, respectively. With sufficient chemotherapeutic doses, oncologists may have the opportunity to reduce the recurrence and prolong survival in patients with cervical cancer.

The prophylactic application of myeloid growth factors during chemoradiotherapy is always concerning. In the 1990s, a randomized trial was designed to determine the efficacy of granulocyte-macrophage colony-stimulating factor (GM-CSF) during chemoradiotherapy in patients with limited-stage small-cell lung cancer [18]. Although the use of GM-CSF significantly reduced the frequency of grade 4 neutrophils (18% vs. 24%, *P* = 0.01), it increased the incidence of grade 3 + thrombocytopenia (54% vs. 12%, *P* < 0.001). Moreover, the rate of non-hematological toxicity was higher in the GM-CSF group than in the naïve group. The CONVERT trial [17] reported a significantly reduced FN rate (10% vs. 22%, *P* = 0.002) in patients who received chemoradiotherapy.

Similarly, the incidence of severe thrombocytopenia increased (28% vs. 15%, *P* = 0.001) in the CONVERT trial [18]. Although the incidence of severe anemia was similar between patients with and without G-CSF, the blood transfusion rate was higher in patients who received prophylactic treatment (51% vs. 31%, *P* < 0.001). The authors of the CONVERT trial speculated that with prophylactic G-CSF, patients received the same dose intensity of chemotherapy in subsequent cycles instead of dose reduction after severe neutropenia, possibly resulting in a higher incidence of other myelosuppression. This may be another reason for our study’s relatively high incidence of severe anemia (36.5%).

Our results showed that non-hematological adverse events were mild and tolerable with the prophylactic use of PEG-rhG-CSF. There was no grade 3–4 acute proctitis or cystitis, and all patients (except one who withdrew consent) completed radiotherapy within 8 weeks. Therefore, PEG-rhG-CSF does not aggravate radiation-related adverse events and can be safely used during chemoradiotherapy. These results are consistent with those of Zou et al.’s study [15], which showed that the PEG-rhG-CSF experimental group had a similar time to complete radiotherapy as the control group (43.55 days vs. 45.22 days, *P* = 0.375). In addition, no significant differences were noted in non-hematological side effects across the groups. The CONVERT trial reported a similar incidence of grade 3–4 acute esophagitis in patients who received and did not receive G-CSF (19% vs. 20%, *P* = 0.821) [17]. Moreover, severe acute pneumonitis was not observed in either group.

PEG-rhG-CSF is a long-effect stimulating factor that only needs to be subcutaneously injected once per cycle, which could attenuate the pain of multiple injections, reduce travel burden, and improve patient compliance compared to G-CSF [19]. Besides, prophylactic use of PEG-rhG-CSF could decrease the use of salvage G-CSF. In our study, salvage G-CSF occured in 34.6% of participants for 11.5% of cycles. The historical data of our center showed that 50% of patients experienced severe netropenia and the acutual proportion of patients who needed salvage treatment exeeded 50% because patients with continuous grade 2 netropenia also needed G-CSF if they intended to receive further chemotherapy. In the context of the coronavirus disease 2019 (COVID-19) pandemic, the advantages of PEG-rhG-CSF have had a profound impact [20]. The overwhelming burden on the healthcare system rendered it difficult to ensure that patients are receiving sufficient supportive care in a timely manner. In such a scenario, the prophylactic application of PEG-rhG-CSF prevented FN-related hospitalization and reduced the frequency of outpatient visits, consequently minimizing the risk of COVID-19 infection.

The present study had several limitations. First, due to the COVID-19 outbreak and strict restrictions on transportation and hospitalization in Beijing, some patients could not return to the hospital to receive their scheduled chemotherapy on time. Therefore, the estimation of the incidence of adverse effects was affected to some extent. Second, this study enrolled patients with grade 2 anemia, which may have resulted in a higher incidence of severe anemia during treatment and a higher rate of delayed chemotherapy. Third, there are some differences between patients on the chemotherapy regimen and technology of brachytherapy. Patients with bulky tumors usually receive large target volume of radiotherapy, which can lead to severe gastrointestinal and genitourinary toxicities and may cause the interruption of treatment. Thus, we scheduled one cycle of induction chemotherapy to reduce tumor volume. During this study, our institution was attempting to implement IGABT. Due to resouces limitation, only half of participants received this new technology at the discretion of clinician. However, these differences may affect the results of hematological toxicities to some extent. Although this was a prospective study, the superiority of PEG-rhG-CSF could not be ascertained because of the lack of a control group. A randomized controlled trial is needed to evaluate the survival benefit of radiotherapy concurrent with doublet chemotherapy with the support of PEG-rhG-CSF in patients with cervical cancer.

## Conclusions

In conclusion, our study demonstrated that the preventive use of PEG-rhG-CSF reduced the incidence of severe neutropenia during chemoradiotherapy and ensured continuous treatment of patients with cervical cancer with good tolerance.

## Data Availability

The data relevant to this article are available from the corresponding authors upon reasonable request.

## Abbreviations

PEG-rhG-CSF: pegylated recombinant human granulocyte colony-stimulating factor
FIGO: International Federation of Gynecology and Obstetrics
ANC: absolute neutrophil count
G-CSF: granulocyte colony-stimulating factor
FN: febrile neutropenia
CCRT: concurrent chemoradiotherapy
RDI: relative dose intensity
NCCN: National Comprehensive Cancer Network
PEG: polyethylene glycol
CTV: clinical target volume
IGABT: image-guided adaptive brachytherapy
CBC: complete blood counts
CTCAE: Common Terminology Criteria for Adverse Events
GM-CSF: granulocyte-macrophage colony-stimulating factor
COVID-19: coronavirus disease 2019

## References

[1] Chen W, Zheng R, Baade PD, Zhang S, Zeng H, Bray F (2016). Cancer statistics in China, 2015. CA Cancer J Clin.

[2] Morris M, Eifel PJ, Lu J, Grigsby PW, Levenback C, Stevens RE (1999). Pelvic radiation with concurrent chemotherapy compared with pelvic and para-aortic radiation for high-risk cervical cancer. N Engl J Med.

[3] Rose PG, Bundy BN, Watkins EB, Thigpen JT, Deppe G, Maiman MA (1999). Concurrent cisplatin-based radiotherapy and chemotherapy for locally advanced cervical cancer. N Engl J Med.

[4] Chemoradiotherapy for Cervical Cancer Meta-Analysis Collaboration (2008). Reducing uncertainties about the effects of chemoradiotherapy for cervical cancer: a systematic review and meta-analysis of individual patient data from 18 randomized trials. J Clin Oncol.

[5] Tan LT, Pötter R, Sturdza A, Fokdal L, Haie-Meder C, Schmid M (2019). Change in patterns of failure after image-guided brachytherapy for cervical cancer: analysis from the RetroEMBRACE study. Int J Radiat Oncol Biol Phys.

[6] Dueñas-González A, Zarbá JJ, Patel F, Alcedo JC, Beslija S, Casanova L (2011). Phase III, open-label, randomized study comparing concurrent gemcitabine plus cisplatin and radiation followed by adjuvant gemcitabine and cisplatin versus concurrent cisplatin and radiation in patients with stage IIB to IVA carcinoma of the cervix. J Clin Oncol.

[7] Ma S, Wang J, Han Y, Guo F, Chen C, Chen X (2019). Platinum single-agent vs. platinum-based Doublet agent concurrent chemoradiotherapy for locally advanced cervical cancer: a meta-analysis of randomized controlled trials. Gynecol Oncol.

[8] Kuderer NM, Dale DC, Crawford J, Lyman GH (2007). Impact of primary prophylaxis with granulocyte colony-stimulating factor on febrile neutropenia and mortality in adult cancer patients receiving chemotherapy: a systematic review. J Clin Oncol.

[9] Lyman GH, Dale DC, Wolff DA, Culakova E, Poniewierski MS, Kuderer NM (2010). Acute myeloid leukemia or myelodysplastic syndrome in randomized controlled clinical trials of cancer chemotherapy with granulocyte colony-stimulating factor: a systematic review. J Clin Oncol.

[10] National Comprehensive Cancer Network. Hematopoietic growth factors. Version 2.2023. https://www.nccn.org/guidelines.

[11] Martínez-Fernández MI, Legaspi Folgueira J, Valtueña Peydró G, Cambeiro M, Espinós J, Aramendía JM (2016). Long-term results of a phase II trial of concomitant cisplatin-paclitaxel chemoradiation in locally advanced cervical cancer. Int J Gynecol Cancer.

[12] Molineux G (2004). The design and development of pegfilgrastim (PEG-rmetHuG-CSF, Neulasta). Curr Pharm Des.

[13] Xie J, Cao J, Wang JF, Zhang BH, Zeng XH, Zheng H (2018). Advantages with prophylactic PEG-rhG-CSF versus rhG-CSF in breast cancer patients receiving multiple cycles of myelosuppressive chemotherapy: an open-label, randomized, multicenter phase III study. Breast Cancer Res Treat.

[14] Liu X, Meng Q, Wang W, Zhou Z, Zhang F, Hu K (2019). Predictors of distant metastasis in patients with cervical cancer treated with definitive radiotherapy. J Cancer.

[15] Zou D, Guo M, Zhou Q (2021). A clinical study of pegylated recombinant human granulocyte colony stimulating factor (PEG-rhG-CSF) in preventing neutropenia during concurrent chemoradiotherapy of cervical cancer. BMC Cancer.

[16] Yanazume S, Karakida N, Higashi R, Fukuda M, Togami S, Kamio M (2018). Tumor bleeding requiring intervention and the correlation with anemia in uterine cervical cancer for definitive radiotherapy. Jpn J Clin Oncol.

[17] Gomes F, Faivre-Finn C, Mistry H, Bezjak A, Pourel N, Fournel P (2021). Safety of G-CSF with concurrent chemo-radiotherapy in limited-stage small-cell lung cancer - secondary analysis of the randomised phase 3 CONVERT trial. Lung Cancer.

[18] Bunn PA, Crowley J, Kelly K, Hazuka MB, Beasley K, Upchurch C (1995). Chemoradiotherapy with or without granulocyte-macrophage colony-stimulating factor in the treatment of limited-stage small-cell lung cancer: a prospective phase III randomized study of the Southwest Oncology Group. J Clin Oncol.

[19] Liu F, Du Y, Cai B, Yan M, Yang W, Wang Q (2017). A clinical study of polyethylene glycol recombinant human granulocyte colony-stimulating factor prevention neutropenia syndrome in patients with esophageal carcinoma and lung cancer after concurrent chemoradiotherapy. J Cancer Res Ther.

[20] Cooksley T, Font C, Scotte F, Escalante C, Johnson L, Anderson R (2021). Emerging challenges in the evaluation of fever in cancer patients at risk of febrile neutropenia in the era of COVID-19: a MASCC position paper. Support Care Cancer.

